# Indigenous knowledge and nutritional and morphological characterization of okra (*Abelmoschus esculentus* (L.) Moecnh) varieties in Western Ethiopia

**DOI:** 10.1002/fsn3.3936

**Published:** 2024-01-03

**Authors:** Hasebu Yassin Mohammed, Tadesse Fikre Teferra, Getachew Sime

**Affiliations:** ^1^ Department of Biology, College of Natural and Computational Sciences Assosa University Asosa Ethiopia; ^2^ School of Nutrition, Food Science and Technology, College of Agriculture Hawassa University Hawassa Ethiopia; ^3^ Department of Biology, College of Natural and Computational Sciences Hawassa University Hawassa Ethiopia

**Keywords:** ethno‐botany, ethnomedicine, indigenous knowledge, indigenous okra, proximate composition, traditional uses

## Abstract

Okra (*Abelmoschus esculentus* (L.) Moecnh) is a versatile crop that is widely grown in western Ethiopia, despite that it is less researched. This study, therefore, investigates the traditional knowledge, nutritional, and morphological characteristics of two commonly grown okra varieties (Kenketse and Sharma) in west Ethiopia. A semi‐structured questionnaire, checklist, and observations were used to collect primary data from households, key informants, and farms. In addition, proximate composition parameters like moisture content, total ash, crude fat, crude protein, crude fiber, utilizable carbohydrates, and gross energy fruits were measured through laboratory analysis. Fruit morphological traits, including fruit dimensions such as length and width, were determined using a digital Vernier caliper. For data analysis, SPSS software was used. The results revealed that the entire plant is used for both food and ethnomedicinal purposes. Curing ailments such as stomach pain, constipation, wound/cut, heart discomfort, back pain, malaria, diarrhea, and amoebae were among the medical benefits of okra. The proximate composition (%) on a dry matter basis of okra fruits differed significantly (*p* < .05) by cultivar type and drying method. Moisture (3.69%–5.24%), total ash (6.60%–8.01%), crude fat (4.87%–8.58%), crude protein (4.06%–5.23%), crude fiber (12.85%–15.26%), utilizable carbohydrate (56.42%–67.63%), and gross energy contents (326–341 kcal) were the ranges of the various proximate and energy levels. Morphological characteristics of fruit and leaf length, width, and length‐to‐width ratio varied with cultivar type, where Sharma had more than double the length of Kenketse (70.1 vs. 187.1 mm). Despite being an undervalued native vegetable crop in Ethiopia, the study's findings show that okra is a multipurpose vegetable for promoting human health and ensuring food and nutritional security. Therefore, stepping up its production, marketing, and consumption could help alleviate the food shortage and improve human nutrition and health in the study areas.

## Graphical abstract

Geometric physical characteristics of two okra fruits showed huge variation in terms of their length, width, and length‐to‐width ratio.

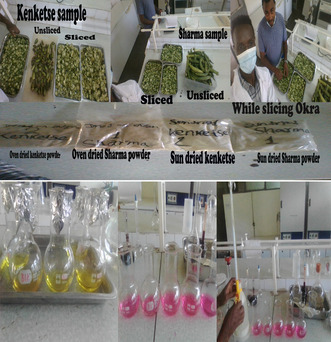

## 1 INTRODUCTION

Plants are used as pharmaceuticals, nutraceuticals, cosmetics, and food supplements, even as traditional sources of medicines and they also play a vital role as the sources of prescription drugs in allopathic medicines (Das et al., 2019). Among plant sources, vegetables play important roles in human nutrition and health (Oguntibeju et al., 2013). This is due to their high levels of vitamins, minerals, dietary fiber, and phytochemicals, in addition to protein and energy (Dias & Ryder, 2011). Commonly, vegetables provide the health‐promoting properties, nutrients, and enzymes necessary for proper body functions (Gemede et al., 2015), while no single vegetable provides all the nutrient requirements (Uusiku et al., 2010; Wallace et al., 2020).

Ethiopia is the home for a high diversity of traditional knowledge and practices about the uses of traditional medicine due to the existence of different ethnic groups and complex cultural diversity Assen et al., 2021). In addition, it has a vast genetic diversity of plant species, including important indigenous vegetables. Some of the indigenous vegetables that are native to Ethiopia include okra (*Abelmoschus esculentus*) in west Ethiopia, jute mallow (*Corchorus olitorius*) in the Afar region, moringa (*Moringa olifera*) in south Ethiopia, and anchote (*Coccinia abyssinica*) in west Ethiopia (Kumar et al., 2013; Simonne et al., 2010). Among these vegetables, okra is a multipurpose crop due to the various uses of its edible parts, like the fresh leaves, buds, flowers, pods, stems, and seeds (Yonas et al., 2014) which are widely consumed in western Ethiopia. The whole plant is edible and has various food, non‐food, and medical applications, and the seed is the nutritionally richest part of the okra (Adelakun et al., 2009). It has long been used as a vegetable and a source of dietary medicines (Benchasri, 2012; Elkhalifa, Alshammari, et al., 2021).

Okra grows preferably in well‐drained, humus‐rich fertile soil with a pH ranging from 6 to 6.7 (Jain et al., 2012), grows in poor soil with intermittent moisture (Ogungbenle & Omosola, 2015), adapts to a wide range of climatic conditions (Akanbi et al., 2010), and grows best in hot weather (temperatures above 26°C) (Ndunguru & Rajabu, 2004). It is an economically important vegetable crop (Ismail & Ibn Idriss, 2013), is widely cultivated throughout the year in the tropics (Singh et al., 2014), and is grown as a garden crop or on large commercial farms (Mokgalabone et al., 2023).

Okra is known by different local names in its area of origin (Benishangul Gumuz Regional State): Kenkatse (Berta), Andeha (Gumuz), and Bamuya (Afaan Oromo) (Gemede et al., 2015), which are some of the popular local names of okra. It is a major traditional vegetable among the Berta community (one of the ethnic groups in the region) and is consumed as both a fruit and a vegetable. It is a subsistence crop commonly produced to fill food shortages throughout all months of the year. The cultivation of okra has strong cultural ties with the Berta communities, since it is used as cultural food during festivals, weddings, and parties and consolidation days. It is also believed that okra makes lactating mothers healthier and stronger (Kushi et al., 2023).

Despite the nativity of okra to Ethiopia and its large importance to local communities for multiple purposes, there are only limited studies to promote and enhance the food value of okra. It is considered as an orphan crop (Kushi et al., 2023). In addition, there is limited market value for okra, partly due to the limited knowledge on the specific variety/genotype for food, non‐food, and medicinal uses. It is an underutilized indigenous plant species in Ethiopia that is a locally available and affordable source of energy and important nutrients (Negash et al., 2023).

There is a lack of awareness and information regarding the importance of okra, particularly its medicinal benefits, morphological features, genetic diversity, nutritional composition, and food value for humans. It is a native plant that is underutilized. Nevertheless, research and development efforts to capitalize on the potential in food and nutrition values, ethnomedicinal effects, and morphological traits of many okra varieties are negligible. Such studies are intended to promote the ethnomedicinal benefits, food and nutritional qualities, and genetic diversity of okra, which will play an important role in alleviating malnutrition, reducing food insecurity, and improving human health and nutrition. Therefore, the overall objective of this research was to look into indigenous knowledge and nutritional and morphological characterization of two okra genotypes in western Ethiopia.

## 2 MATERIALS AND METHODS

### 2.1 Description of the study site

**FIGURE 1:**
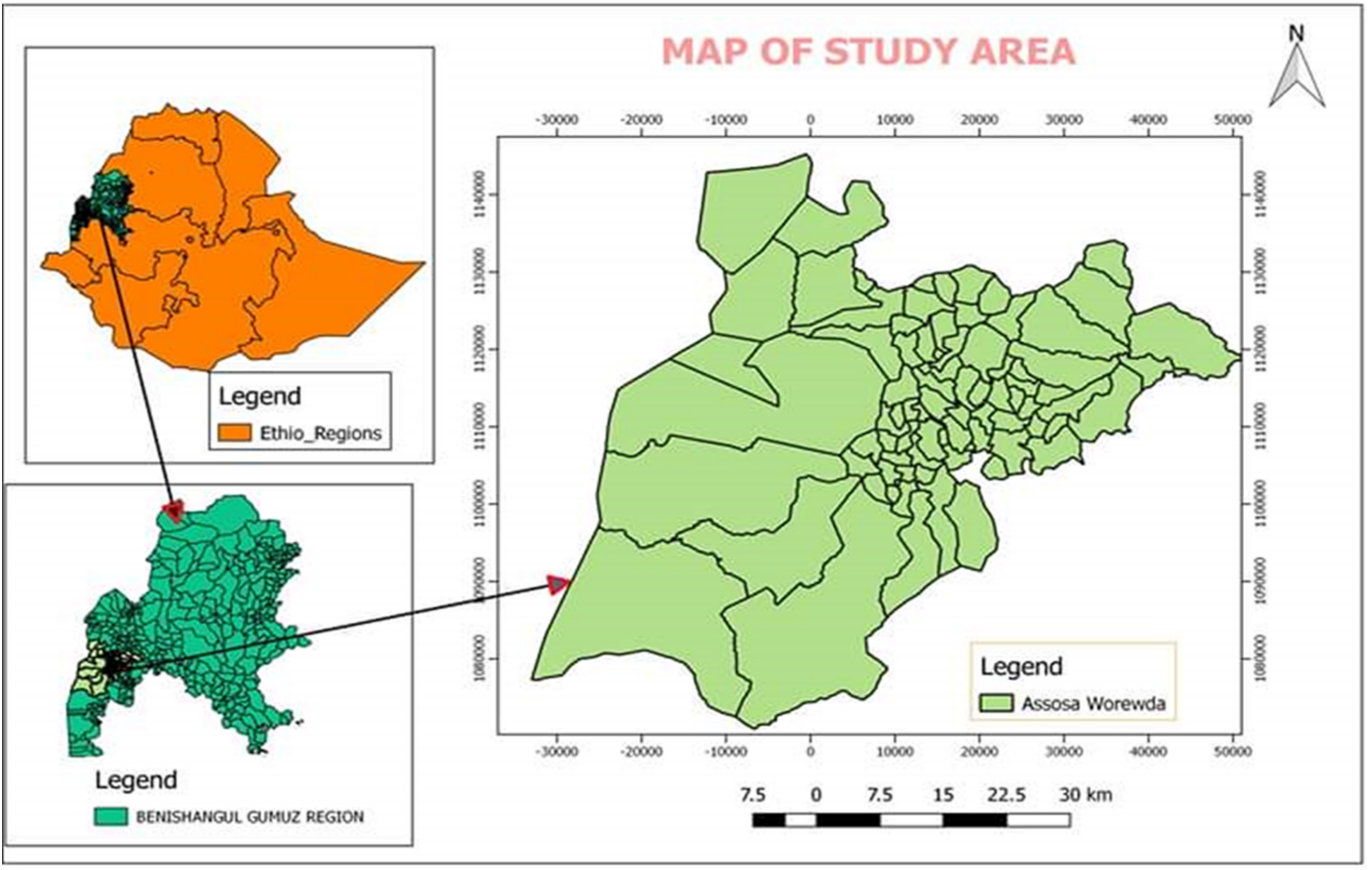
Map of the study area.

The research site is in Benishangul Gumuz Regional State (Figure 1). It is located 661 km west of Ethiopia's capital city, Addis Ababa. It is located at 34,012′32″ N and 10,035′45″ E. According to the Population and Housing Census of Ethiopia in 2007, the total population was estimated to be 87,366 (44,176 males and 43,190 females), with approximately 74% residing in rural regions. It had a population density of roughly 37.7 people per km^2^ and covered 2371 km^2^.

### 2.2 Agro‐ecology, climate, and vegetation

The climate is humid and hot, with distinct dry and wet seasons. The annual average temperature and total annual rainfall range from 15.4°C to 29.6°C and 1132.9 mm, respectively. In general, the climate is favorable for both annual and perennial crops (Tamrat et al., 2016). It is divided into two traditional climatic zones based on altitudinal variation: lowlands and midlands. The district's geography is hilly (30%), plain (52%), and depression (18%). The elevation varies between 558 and 2729 m above sea level. It is characterized by rugged terrain and plain surfaces. The study site has a variety of vegetation due to variations in soil, temperature, and human activity. The area contains dense vegetation, with tropical bamboo forest, acacia and other trees, tall grasses, herbs, bushes, and shrubs (Mohammed, 2010).

### 2.3 Data collection methods

Two methods of data collection were used: (1) Ethno‐botanical data on the food value and medicinal benefits of okra, as well as an assessment of the physical characteristics of the two most commonly utilized okra varieties (Kenkatse and Sharma), were collected. This was accomplished by conducting a household survey utilizing a questionnaire and key informant interviews, and (2) collecting data on the nutritional quality of two commonly produced okra fruits for laboratory examination. The nutritional composition of Kenkatse and Sharma okra fruits was examined using electric drying and sun drying methods.

#### 2.3.1 Techniques for selecting study sites, households, and key informants

The study site was chosen using the purposive sampling technique since it is in an area where the okra plant is commonly cultivated and used for a variety of reasons. Simple random sampling procedures were used to pick 164 sample households from three typical villages. The adoption of a simple random sample size was justified due to the homogeneity of the households in terms of socio‐cultural and economic elements, as well as the representation of the research communities that widely cultivate okra. In addition, nine key informants were chosen from each of the three communities using a purposive sampling technique (Table 1). Those who were familiar with indigenous knowledge of using okra as food, traditional medicine, and plant parts used as food and medicine were key informants. They were agricultural specialists, herbalists, elders, and model farmers. The key informants were chosen on the advice of elders and local officials (development agents and village administrative leaders).

**TABLE 1:** Study village, sample village, and sample respondents.

Study village	Household	Key informant	Total	%
*Buldigilu*	37	9	46	23
*Nebar Komoshiga*	53	9	62	31
*Gambeshiri*	29	9	38	19
*Tsentsehallu*	45	9	54	27
Total	164	36	200	100

Data from 164 households was acquired through household head interviews utilizing semi‐structured questionnaires (Table 1). The key informant interviews were conducted using a questionnaire checklist.

Yamane (1967) was used to determine the sample size.

$$n=N/1+N\;\left(e\right)\;2$$

where *n* is the sample size, *N* is the population size, and *e* is the acceptable sampling error.

#### 2.3.2 Plant part sampling and laboratory analysis (Figures 2, 3, 4)

**FIGURE 2:**
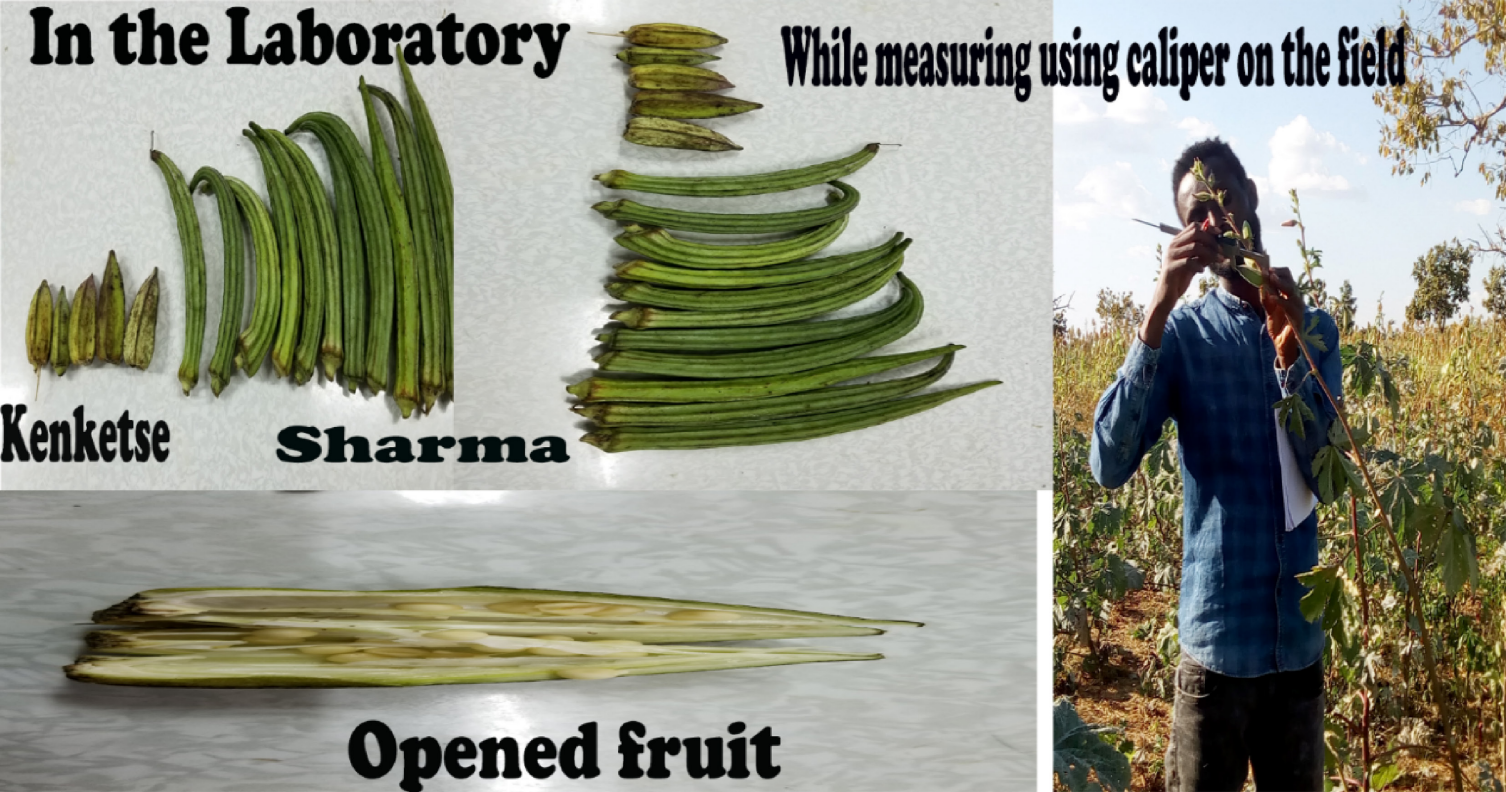
Pictures while measuring okra pods on the farm and pods of two cultivars in the laboratory.

**FIGURE 3:**
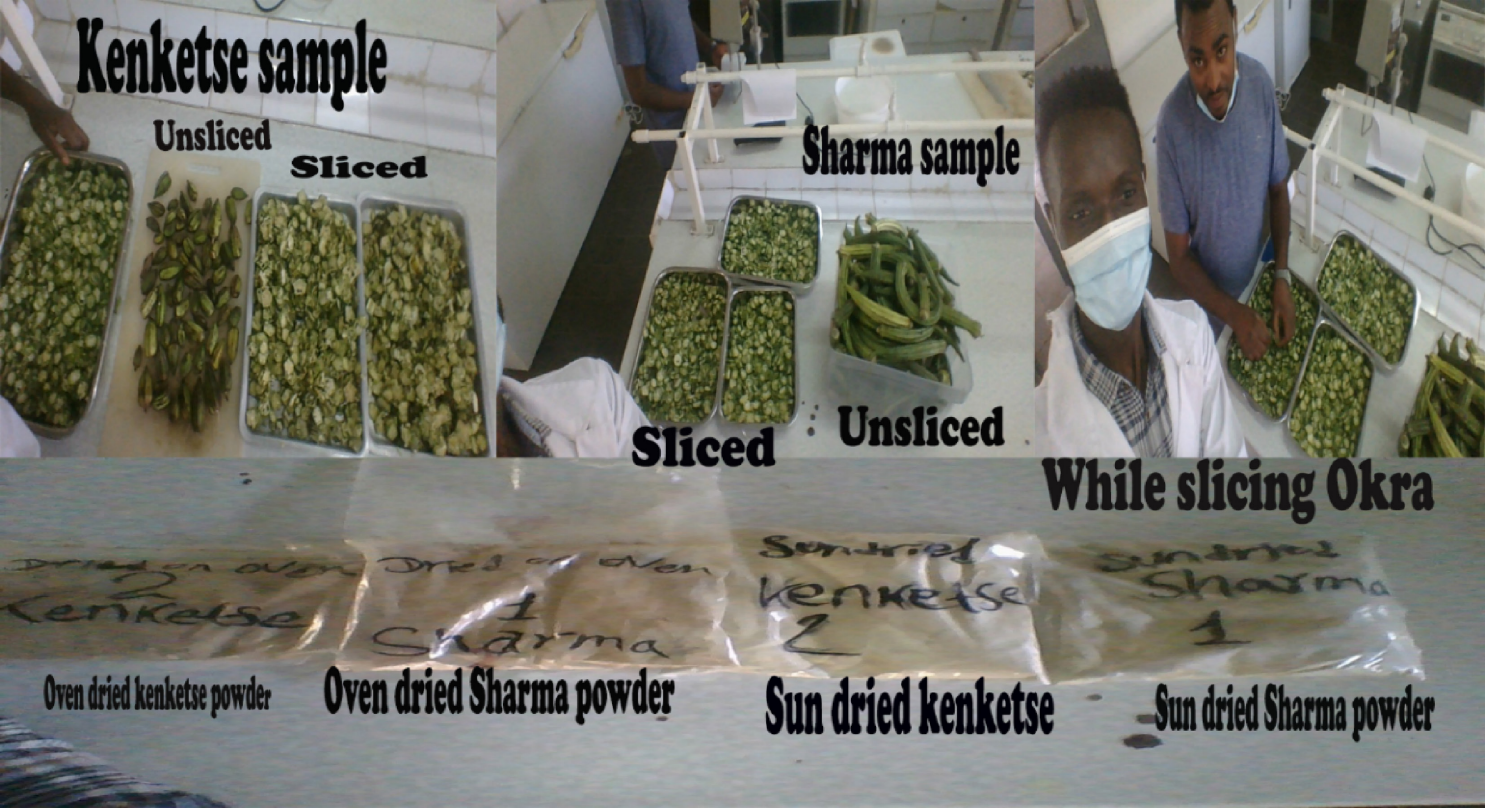
Slicing, drying, and powdering samples of Kenketse and Sharma.

**FIGURE 4:**
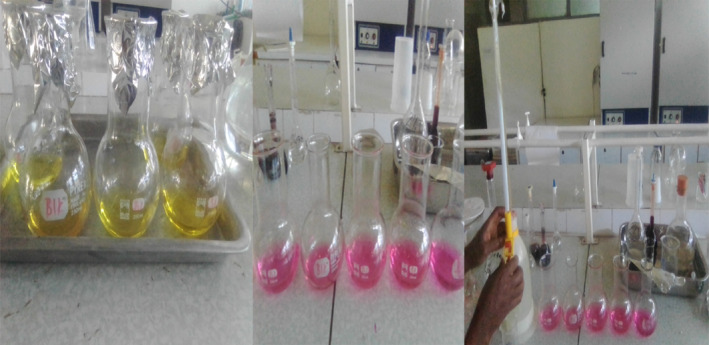
Digestion, distillation, and titration of samples of Kenketse and Sharma.

##### Sampling procedures

Fresh (completely immature fruit) samples of okra were randomly collected from farms. The fresh okra fruits were then dried and ground in the same agricultural areas.

##### Laboratory analysis of the nutritional compositions of okra

After collecting samples of the two most common types of okra pods, the proximate composition (such as moisture, crude protein, fat, ash, crude fiber, utilizable carbohydrate, and gross energy) of the fruits was analyzed using AOAC (AOAC, 2000) protocols.

##### Determination of moisture content

The moisture content of two okra cultivars' pod samples (Kenketse and Sharma) was measured using standard methods (AOAC, 2000) sub‐component 925.09 by oven drying. A clean aluminum dish was dried in a drying oven (DHG‐9055A) at 105°C for 1 h, then chilled in a desiccator (CSN‐SIMAX) for roughly 30 min before being weighed.

The samples prepared in duplicate for each treatment were thoroughly mixed, and approximately 10 g and 3.0 g of samples on a dry basis were weighed. The pre‐weighted plates and their contents were dried for 12–16 h at 85–100°C in the drying oven. After drying, the samples were cooled in a desiccator for 30 min before being re‐weighed to achieve a constant weight. The moisture content was estimated using the formula below:

$$\mathrm{MC}\;\left(\%,\mathrm{db}\right)=\frac{\left(W2-W1\right)-\left(W3-W1\right)}{\left(W3-W1\right)}*100$$

where MC = moisture content, *W*1 = weight of empty crucible, *W*2 = weight of crucible with fresh sample, *W*3 = weight of crucible with dried sample.

##### Determination of total ash content

The total ash content of pod (Kenketse and Sharma) samples was determined using established methods (AOAC, 2000) by burning known weights of the samples in a muffle furnace (Carbolite CSF 1200) at 550°C until a white ash was formed. Each dish received 3.0 g of material from each treatment in duplicate. The samples were placed in the muffle furnace (Carbolite CSF 1200) and incinerated for 3 h at 550°C. The samples were extracted from a Muffle Furnace and cooled; they appear clean and white. A few drops of de‐ionized water and strong nitric acid were added, dried, and returned to a muffle furnace, where they were tested for evidence of carbon. Finally, the samples were removed from the muffle furnace and immediately placed in a desiccator to cool to ambient temperature, and each dish plus ash was reweighed. Using the following formula, the weight of total ash was computed by difference and expressed as a percentage of samples:

$$\mathrm{AC}\;\left(\%,\mathrm{db}\right)=\frac{W2-W1}{W3}*100$$

where AC = ash content, W1 = weight of empty crucible, W2 = weight of crucible with ash, W3 = weight of sample.

##### Determination of crude fat content

The crude fat content of okra pod samples (Sharma and Kenketse) was determined using standard procedures (AOAC, 2000) and subcomponent 920.39 in a Soxhlet extractor. The cleaned extraction flasks with boiling chips were dried in Oven Drying (DHG‐ 9055A) for 1 h at 90°C, cooled in desiccators (with granular silica gel desiccants) for 30 min, and weighed. A 2 cm layer of fat‐free cotton was placed on the bottom of the extraction thimble. In the extraction thimbles, 3.0 g of okra samples from each treatment (raw or control, boiled after peeling, and boiled before peeling) were placed in triplicate and then coated with a 2 cm layer of fat‐free cotton.

The sample‐filled thimbles were put in the Soxhlet extraction chamber (Shanghai Qianjian Instrument Co., Ltd.). The cooling water was turned on, and 500 mL of diethyl ether was introduced into the extraction flask via the condenser. The extraction lasted roughly 4 h. The extraction flasks were withdrawn from the extraction chamber and placed in the drying oven at 90°C for about 30 min, then cooled to room temperature in the desiccator for about 30 min before re‐weighing the flask with the extract.

##### Determination of crude protein content

The protein content of pod (Kenketse and Sharma) samples was determined using conventional methods (AOAC, 2000) by the Kjeldahl method, which included digestion, distillation, and titration, as follows:

###### Digestion

A small digestion flask (250 mL) was filled with 0.2 g of the material. Two catalyst tablets (anhydrous sodium sulfate + copper sulfate) were added to the sample, along with 3.5 mL of 98% H_2_SO_4_. The flask's contents were then heated on an electrical heater for 2 h until the color changed to blue‐green. After that, the tubes were withdrawn from the digester and left to cool.

###### Distillation

The digested sample was passed to the distillation unit along with 15 mL of 40% NaOH. The ammonia was placed in a 100 mL conical flask with 10 mL of boric acid and 3–4 drops of methyl red indicator. The distillation process was repeated until the volume reached 50 mL.

###### Titration

The flask's contents were titrated against 0.1 N HCl. The titration reading was noted. The crude protein was determined using the equation below:

$$\mathrm{CP}\;\left(\%,\mathrm{db}\right)=\frac{\left(T-B\right)*N*14*100*6.25}{\mathrm{WS}*1000}$$

where CP = crude protein, *T* = titration reading, *B* = blank titration reading, *N* = normality of HCl, WS = weight of sample, 1000 = to convert to mg, 6.25 = protein factor.

##### Determination crude fiber contents

The crude fiber content was measured using conventional procedures (AOAC, 2000). Approximately 1 g of defatted pod samples were treated sequentially with boiling solutions of H_2_SO_4_ (0.26 N) and KOH (0.23 N). The residue was then filtered, washed, and put in a crucible, which was then placed in an oven set to 105°C for 18–24 h. The sample‐filled crucible was weighed and incinerated in a muffle furnace at 500°C. The crude fiber was determined using the equation below:

$$\mathrm{FC}=\frac{W2-W1}{W3}$$

where CF = crude fiber, *W*1 = weight of crucible with sample before incineration, *W*2 = weight of crucible with sample after incineration, *W*3 = weight of sample.

##### Determination of utilizable carbohydrates

Utilizable carbohydrate (UC) content was calculated using the mathematical expression as follows:

$$\%\mathrm{UC}=100–\left(\%\text{moisture}+\%\text{crude protein}+\%\text{crude fiber}+\%\text{total}\;\mathrm{ash}+\%\text{crude}\;\mathrm{fat}\right)$$

##### Determination of gross energy

The percent calories in the chosen samples were determined by multiplying the crude protein and carbohydrate percentages by 4 and the crude fat percentage by 9. The results were then converted to kilocalories per 100 g of sample.

##### Determination of the morphological characteristics of okra

To establish the mean dimension of the pods, 17 replications of each cultivar (Kenketse and Sharma) were chosen at random (Figure 2). The length (*L*) and width (*W*) of the pods were measured using a digital Vernier caliper with an accuracy of 0.01 mm. The two axial dimensions were used to calculate the mean length of pods and seeds. The length was chosen as the largest dimension, and the width was picked as the shortest. The arithmetic mean diameter (Da) and geometric mean diameter (Dg) were computed using the relationships shown below (Fadavi et al., 2013). The *D*_a_ and *D*_g_ values were calculated using the following formula (Equations 1 and 2):

(1) $$D_{a}=L+W/2$$

(2) $$D_{g}={\left(\mathrm{LW}/2\right)}^{0.5}$$

### 2.4 Data analysis

#### 2.4.1 Ethno‐botanical data analysis

An Excel spreadsheet was utilized for descriptive statistics, such as percentage, frequency distribution, and graphs, and SPSS software (version 20) was utilized for the analysis of ethno‐botanical data generated through semi‐structured questionnaires.

#### 2.4.2 Informant consensus

To test the reliability of information during the interview, respondents were contacted at least twice for similar responses, and the validity of the information was proved and recorded. When a respondent's information deviates from the initial notion, it is discarded because it is deemed untrustworthy (Alexiades, 1996; Seddig & Leitgöb, 2018).

#### 2.4.3 Experimental data analysis

A factorial design was employed for the proximate study of the two types of okra in both fresh and dried variants. All experiments for the chemical analysis were duplicated, while the physical attributes were reproduced 17 times. The data was analyzed with a statistical tool (SAS software) and an analysis of variance (ANOVA). To separate the means, Fisher's least significant difference (LSD) was applied, and the results were reported as mean with least squares means with standard errors.

## 3 RESULTS AND DISCUSSION

### 3.1 Demographic characteristics

Age, gender, educational status, occupation, marital status, income level, and ethnicity were among the socioeconomic characteristics of the sample households studied. In terms of gender, the number of female‐headed households (57.5%) was higher than that of their male‐headed counterparts (42.5%). The dominance of female‐headed households is attributed to their cultural attachment to the plant from seedbed preparation through harvesting, owing to the customs and beliefs of the local communities. The majority of households (44%) had an age range of 18–40 years, followed by 41–60 years. In terms of educational status, the majority of households (40.5%) had formal primary education, while 32.5% were illiterate. Farming (72.5%) was the most common occupation, followed by engaging in traditional healing (11%). The majority of households (55.5%) were classified as medium‐income, as high‐income (24.5%), and as low‐income (20%).

### 3.2 Ethno‐botanical status, cultivars, cultivation purposes, and growing season

The cultivated herb known as “Kenketse” was familiar to all of the households. The majority of households (98%) used two local names for the two main cultivars, Kenketse adaru and Sharma. Most of them (70%) used three sub‐cultivars of “Kenketse adaru,” namely Kenketse bertha, Kenketse futie, and Kenketse begnenie, while 17.9% used only Kenketse futie and 1% used only Kenketse begnenie. Likewise, the majority of households (68.5%) used two sub‐cultivars of “Sharma,” namely Shrama dhugurie and Sharma tsurignie, where 28% used Sharma dhugurie and the remaining 3.5% used Sharma tsurignie. This demonstrates that the majority of households consume several okra sub‐cultivars.

**FIGURE 5:**
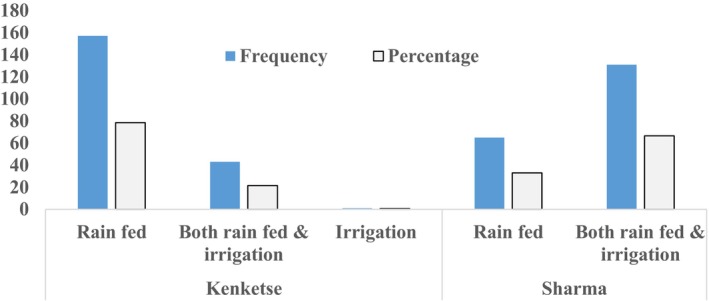
Growing seasons of two main cultivars of okra.

In accordance with consumption preferences, a small percentage of households grew only Kenketse adaru, while the vast majority of households grew both cultivars (Kenketse adaru and Sharma). Okra was grown by every household mainly for food and income sources as well as ethnomedicine. Regarding production seasons, although it was grown in both the rainy and irrigated seasons, Kenketse adaru was mostly grown in the rainy season. In contrast, Sharma was primarily grown in both irrigated and rain‐fed systems, though some households only grew it in irrigation or rain‐fed systems (Figure 5). This shows that okra is grown all year round, suggesting that it may help ensure food security, generate income, and have ethnomedical benefits. Previous studies also demonstrate okra to be an important vegetable crop with a diverse array of nutritional qualities and potential health benefits (Das et al., 2019; Elkhalifa, Alshammari, et al., 2021).

Concerning life span, Sharma was the fastest‐growing okra cultivar, particularly Sharma dhugurie, which completes its life cycle in 40–50 days, whereas Sharma tsurignie completes its life cycle in 60–80 days. Kenketse adaru, on the other hand, takes 120–190 days from planting to harvesting. This shows that, compared to the Kenketse cultivar, Sharma can improve food security and generate revenue more quickly. More significantly, cultivating various cultivars with distinct life cycles could guarantee that the benefits are available all year long.

### 3.3 Traditional uses and edible parts

The traditional utilization and consumption of okra are mostly tied to traditional knowledge and socio‐cultural practices. It was attested that the different parts of the plant are edible: pod (100%), seed (99%), flower (83%), leaf (65.5%), and root (28%) (Table 2). This illustrates that every part of the plant is edible and economically important to the local community. The seeds can also be used as a coffee substitute. Stems are also used as fodder for livestock and as fuel to cook food. Aside from that, because the stem's rough hairs instantly kill snakes upon contact, it has historically been used to keep snakes out of houses. This result is in line with previous studies showing that the different parts of okra are edible and provide additional cooking and medicinal benefits (Elkhalifa, Alshammari, et al., 2021; Romdhane et al., 2020).

**TABLE 2:** Edible parts of okra.

No.	Edible plant parts	Households		%
		Response	Frequency	
1	Leaf	Yes	137	68.5
		No	63	31.5
2	Flower	Yes	166	83
		No	34	17
3	Seed	Yes	199	99.5
		No	1	0.5
4	Pod/Fruit	Yes	200	100
		No	0	0
5	Root	Yes	56	28
		No	144	72

#### 3.3.1 Mode of preparation and uses as food

Okra is a versatile vegetable that can be used to prepare a variety of dishes. In addition to food, the pod is used for making luxury drinks, such as soup, coffee, juice, and others, based on the communities' desires and experiences. The pod is the most useful part of the crop, serving several functions:

##### Stew (wat) preparation

Stew preparation “draw” is the most popular mode of preparation of okra, where all parts are processed either in fresh or dry powdered forms. To prepare stew, fresh leaves, flowers, bark, and pods are harvested, well‐washed, and ground to make dry powders. It is then mixed with additional ingredients such as boiled split beam, red pea, green pea, black pea, split pea, soybean, mung bean, split mung, and water before being heated until well‐cooked and spiced/seasoned. Sew is typically served with local stable bread, such as *injera*, *aljizira*, *alguraza*, or porridge. The mucus‐like liquid is used to thicken strews, which are popular among the local community, as also reported previously (Adiaha, 2017; Goksen et al., 2023).

##### Soup preparation

The popular “draw” (sticky) soup, also known as “kenketse soup,” is often cooked with either red meat or alone (Gemede et al., 2018). To make soup, immature fresh “kenketse bertha” is picked, washed, chopped, or sliced into tiny sizes, then combined with additional ingredients, such as ground red meat, sliced carrot, sliced onion, oil, dried fish, and water. It is then boiled for half an hour to preserve the volatile nutrients. The soup is utilized as a fast‐track to meet the immediate requirement for food by locals who have been hungry for several hours.

##### Salad preparation

The pods are chopped and boiled with pepper to make okra salad, which is served with local foods such as *injera*, *aljizra*, and *alguraza*, which are made of powdered corn, sorghum, *teff*, or rice. This demonstrates that the pod can be consumed as a vegetable, either raw or cooked. The local communities also utilize okra seeds as a coffee replacement when there is a coffee scarcity or lack of access to coffee (Gemede et al., 2015).

### 3.4 Ethnomedicinal uses

**FIGURE 6:**
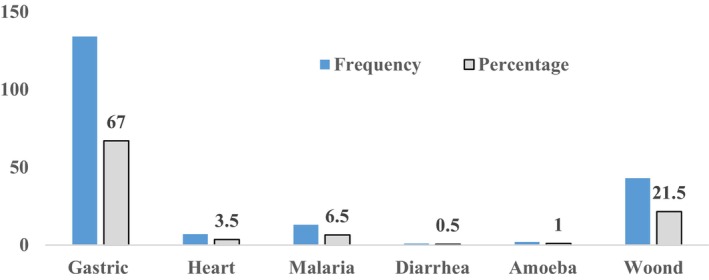
Okra‐treated diseases in the research areas.

Most households utilized the entire plant to heal or treat a variety of diseases, including back pain, heart discomfort, constipation, gastric infection, malaria, diarrhea, amoeba, and many sorts of wounds (Table 3). A larger number of households (55.5%) used all parts of the plant, while 27.5% used pods, 11% used seeds, 3.5% used flowers, 1.5% used barks/resins, and 1% used leaves to treat various diseases (Figure 6).

**TABLE 3:** Ethnomedicinal Benefits of Okra in the Study Areas.

No.	Disease	Plant part used	Method of preparation
1	Gastric disease	Flower, pod, leaf, and bark	Decoction or soup
2	Constipation	Fresh seed	Raw
3	Wound healing/cut	Flower, pod, leaf, and bark	Crushed raw
4	Heart pain	Fresh seed	Raw
5	Malaria	Dry seed	Juice of okra mixed with cardamom and prepared as infusion
6	Diarrhea	Pod	Powdered and mixed with powdered maize prepared as soup
7	Amoebae	Root	Infusion
8	Back pain	Fresh pod	Decoction

The different parts of the plant were utilized to treat various ailments (Tables 3 and 5; Figure 5). Gastric infection, constipation, wound/cut, and heart ache were among the common ailments healed by the plant. It is also highly recommended for the treatment of severe diabetes patients. Okra is a multifunctional crop for treating both human and livestock ailments (Abd El‐Hack et al., 2018; Uba et al., 2022).

**FIGURE 7:**
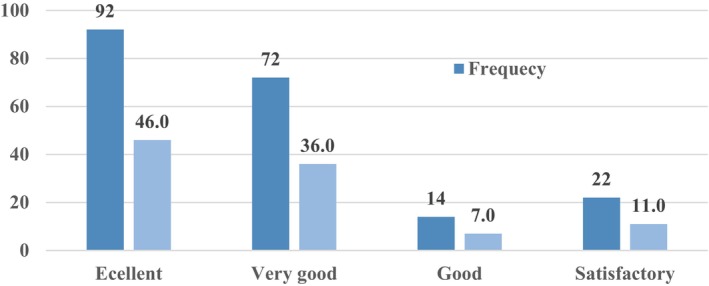
The effectiveness of okra in treating human ailments.

Regarding effectiveness in treating ailments, most households ranked okra as excellent, while 36% ranked it as very good (Figure 7). This suggests that the plant is efficient in treating a variety of illnesses. It provides the local community with cultural value in addition to healing a variety of ailments.

### 3.5 Proximate composition

#### 3.5.1 Moisture content

Table 4 shows the moisture content of two okra cultivars dried by both sun and oven drying methods. Moisture content is the amount of free water present in a given food sample on a wet or dry basis. The moisture content of foods is related to their shelf life. The water content of fresh okra pods varied substantially. To ensure data consistency, the moisture content was estimated on a dry matter basis. The oven and sun‐dried Kenketse had average moisture contents of 2.45% and 6.90%, respectively, whereas Sharma had average moisture contents of 3.69% and 5.24%, respectively (Table 4). Regardless of variant, sun‐dried okra pod samples had significantly (*p* < .05) higher moisture levels than oven dried okra pod samples. This reveals that sun drying, a traditional practice among local communities, becomes more appropriate for having a higher moisture content that is likely in equilibrium with the environment. The moisture content of oven‐dried samples was 2.45% for Kenketse and 3.69% for Sharma, which is higher compared to the earlier report by Kouassi et al. (2013) for Baoule and Dioula varieties (2.50%). Similarly, the moisture contents of sun‐dried Kenketse (6.90%) and Sharma (5.24%) were lower than those reported for Baoule (7.28%) and Dioula (7.33%) varieties by Kouassi et al. (2013). Furthermore, dried powdered Kenketse and Sharma had a longer shelf life, allowing for longer storage and consumption. Okra is highly perishable due to its quick metabolic activities; hence, it must be dried for prolonged storage and use (El‐Shaieny et al., 2022; Falade & Omojola, 2010).

**TABLE 4:** Proximate composition of okra pod by cultivar and drying methods.

Cultivar	MC (db%)	TA (%)	CFat (db%)	CPr (db%)	CF (db%)	UCa (db%)	GE (cal.)
Kenketse, Oven	2.45d	8.01a	4.87c	4.06b	14.62b	65.99b	324.08b
Kenketse, Sun	6.90a	6.60b	8.58a	5.23a	15.89a	56.80c	325.31b
Sharma, Oven	3.69c	7.31ab	6.66b	2.18c	12.49d	67.68a	339.36a
Sharma, Sun	5.24b	7.05b	4.92c	2.01c	13.21c	67.58a	322.63b
SE	0.18	0.19	0.31	0.06	0.011	0.31	1.73

*Note*: Standard error (SE) and means in the same row with different letters are significantly different (*p* < .05).

Abbreviations: CF, crude fiber content; CFat, crude fat content; CPr, crude protein; GE, gross energy content; MC, moisture content; TAs, total ass content; UCa, utilizable carbohydrate.

#### 3.5.2 Total ash content

Table 4 presents the total ash content of the Kenketse and Sharma pods. The average ash concentrations of oven‐ and sun‐dried Kenketse were 8.01% and 6.60%, respectively, while those of Sharma were 7.31% and 7.05%. The average ash level of both cultivars was higher in oven‐dried okra samples than in their sun‐dried counterparts. The ash content of the oven‐dried Kenketse pod was significantly (*p* < .05) higher than the sun‐dried versions for both the Kenketse and Sharma cultivars. Oven drying yielded more ash than sun drying, despite the difference in mean moisture content. The mean ash content of oven‐dried Kenkeste and Sharma was 8.01 g/100 g and 7.31 g/100 g, respectively, which is consistent with the levels reported by Adetuyi et al. (2011). Ash represents the inorganic residues in plant biomasses, thus showing the level of mineral residues such as Ca, Mg, K, Na, Fe, Zn, P, and Cl (Odzijewicz et al., 2022; Tiroesele et al., 2013). High mineral content in foods promotes growth and development by catalyzing metabolic processes in the human body.

#### 3.5.3 Crude fat content

The mean crude fat concentrations in pods of both cultivars after drying were 4.87% and 8.58% for oven‐ and sun‐dried Kenketse and 6.66% and 4.92% for oven‐ and sun‐dried Sharma cultivars, respectively (Table 4). The crude fat content of the pods of both okra cultivars differed significantly (*p* < .05). After sun drying, Kenketse had a greater crude fat level than Sharma, whereas Sharma had a higher crude fat content than Kenketse after oven drying. This demonstrates that the mean crude fat content of pods varied according to the variant and drying technique. The difference is likely due to the proportion of moisture in the final dried specimens that dictates the other constituents. The crude fat content of okra pods for both Kenketse and Sharma was relatively higher than the fat content reported earlier in the literature (Gemede et al., 2015; Kouassi et al., 2013; Nwachukwu et al., 2014). Nonetheless, the mean crude fat content of Kenketse and Sharma was lower than the values reported by Adetuyi et al. (2011). The presence of crude fat concentrations suggests that okra could be used to improve the palatability of foods. There are similar reports from previous studies (Agomuo et al., 2022; Elkhalifa, Alshammari, et al., 2021).

#### 3.5.4 Crude protein content

Kenketse had a significantly (*p* < .05) higher mean crude protein content than Sharma (Table 4). In Kenketse, sun drying favored the crude protein content, while it was oven drying that improved the crude protein in Sharma. The mean crude protein levels for oven‐ and sun‐dried Kenketse were 4.06% and 5.23%, while those of Sharma were 2.18% and 2.01%, respectively (Table 4). This demonstrates that Kenketse had a higher mean crude protein content than Sharma, regardless of drying procedures. The mean crude protein contents of Kenketse (4.65%) and Sharma (2.10%) pods were considerably lower than the values reported for okra pods in previous reports (Adetuyi & Adelabu, 2011; Gemede et al., 2015; Ogungbenle & Omosola, 2015; Sharma & Prasad, 2010). Nonetheless, the mean crude protein level of Kenketse (4.65%) was comparable with the 4.81% reported for okra pods by Nwachukwu et al. (2014). The disparity between our findings and earlier findings might be attributable to variations in the moisture proportions owing to the different drying techniques as well as differences in cultivars and agro‐ecological conditions. Okra is a good source of protein that can help with protein deficiency among local communities and beyond as a source of plant proteins that are trending in recent days.

#### 3.5.5 Crude fiber content

Regardless of drying procedures, the total crude fiber content of Kenketse and Sharma varied significantly (*p* < .05). Kenketse had a significantly higher total crude fiber content (*p* < .05) than Sharma. Moreover, regardless of variants, sun drying yielded significantly higher total fiber content (Table 4). Findings from previous studies also reported variations among different varieties of leaves of fifteen anchote (*Coccinia abyssinica*) accessions (Gemede et al., 2021). The mean value of crude fiber contents recorded for Kenketse (15.26%) and Sharma (12.85%) was generally higher than previous findings reported in the literature (Adetuyi et al., 2011; Kumar et al., 2013; Nwachukwu et al., 2014). However, the mean fiber content of okra is comparable with the results reported by Hussain et al. (2009). Furthermore, the fiber content is higher than that of many regularly consumed vegetables, including sweet potato leaves (7.20%) (Antia et al., 2006), *Allium sativum* bulbs (2.13%), and *Zingiber officinale* rhizomes (2.64%) (Rosc et al., 2018).

This particular finding shows that both okra cultivars could be a good source of dietary fiber, which has a lot of health and nutrition benefits. Nearly half of the okra pod contains soluble fiber in the form of polysaccharides and pectin, which helps lower serum cholesterol and hence the risk of coronary heart disease (Jenkins et al., 2005). The other significant component is the insoluble fiber, which helps to maintain the intestinal tract healthy and reduces irritable bowel syndrome (Yusuf et al., 2022). Moreover, the high fiber content of the okra pod can improve its digestion and absorption processes in the large intestine, stimulating peristalsis and reducing constipation (Gemede et al., 2016).

#### 3.5.6 Utilizable carbohydrate

The mean utilizable carbohydrate content of Sharma was significantly (*p* < .05) higher than Kenketse (Table 4). Moreover, the utilizable carbohydrate content of oven‐dried Kenketse was significantly (*p* < .05) higher than its sun‐dried version (Table 4). This shows that oven drying methods, as opposed to sun drying methods, may have a greater effect on the amount of utilizable carbohydrate in okra. On top of that, the utilizable carbohydrate content varied according to variant and drying process. Kenketse and Sharma had mean utilizable carbohydrate contents of 61.395% and 67.63%, respectively, that coincide with the findings of Hussain et al. (2009), which was 62.05%, and Gemede et al. (2015), which ranged from 56.42% to 69.92%. This demonstrates that okra is a good source of carbohydrates for humans and may meet the necessary nutritional value in the human diet.

#### 3.5.7 Gross energy content

The gross energy content of okra pods varied with cultivars and drying techniques (Table 4). In accordance, oven‐dried Kenketse pods had significantly (*p* < .05) higher gross energy content. Generally, the oven drying method resulted in a slightly higher gross energy content than the sun drying method. This result is consistent with previous studies (Goud et al., 2019; Nwakuba et al., 2020). The mean gross energy content of Kenketse and Sharma was 324 and 330 kcal, respectively, which is higher than the finding reported by Gemede et al. (2015) on eight okra pod accessions that ranged from 257 to 319 kcal and lower than the finding previously reported by Kouassi et al. (2013) of two okra varieties of Bacoule (340 kcal) and Dioula (342 kcal). Variants, drying methods, and agro‐ecological conditions may have contributed to the variations in gross energy content between the findings from the present study and earlier results. The high gross energy value observed in the current study indicates that both cultivars of okra pods could be a rich source of energy, which agrees with results from previous studies (Nwakuba et al., 2020).

Table 5 presents the average proximate composition of okra pods by drying methods. The mean moisture, total ash, crude fat, crude protein, crude fiber, utilizable carbohydrate, and gross energy contents differed significantly (*p* < .05) depending on drying technique and cultivar type. More precisely, when compared to overdrying, the sun drying technique significantly (*p* < .05) increased the mean moisture, crude fat, crude protein, and crude fiber levels. Similarly, when compared to sun drying, oven drying significantly (*p* < .05) increased the mean total ash, utilizable, and gross energy contents (Table 5). This indicates that the cultivar type and drying method have an impact on the proximate composition of okra pods. There are similar results from earlier studies (Goud et al., 2020; Romdhane et al., 2020).

**TABLE 5:** Average proximate composition of okra pods by drying methods.

Drying method	MC (db%)	TA (%)	CFat (db%)	CPr (db%)	CF (db%)	UCa (db%)	GE (cal.)
Oven	3.07b	7.66a	5.77b	3.12b	13.55b	66.834a	331.72a
Sun	6.07a	6.83b	6.75a	3.62a	14.55a	62.19b	323.97b
SE	0.13	0.14	0.22	0.04	0.008	0.221	1.222

Abbreviations: CF, crude fiber content; CFat, crude fat content; CPr, crude protein; GE, gross energy content; MC, moisture content; SE, standard error; TA, total ass content; UCa, utilizable carbohydrate.

Except for the mean moisture and total ash contents, the proximate composition parameters and gross energy contents vary substantially (*p* < .05). Kenketse had significantly higher crude fat, crude protein, and crude fiber contents than Sharma (*p* < .05). Sharma, on the other hand, had significantly (*p* < .05) higher utilizable carbohydrate and gross energy contents (Table 6). This demonstrates that the mean moisture, total ash, crude fat, crude protein, crude fiber, utilizable carbohydrate, and gross energy contents varied with the cultivar types. This result is supported by findings from previous studies by Gemede et al. (2016) in okra and by Adane et al. (2013) in Taro (*Colocasia esculenta* L.). The okra pod's proximate composition demonstrates that it is full of nutrients, which are useful components that have the potential to modulate human physiology and reactions (Agregán et al., 2023).

**TABLE 6:** Proximate composition (db%) of okra pods by cultivars.

Cultivars	MC (db%)	TA (%)	CFat (db%)	CPr (db%)	CF (db%)	UCa (db%)	GE (cal.)
Kenketse	4.67a	7.31a	6.73a	4.65a	15.26a	61.39b	324.70b
Sharma	4.46a	7.18a	5.79b	2.10b	12.85b	67.63a	330.99a
SE	0.13	0.14	0.217	0.04	0.008	0.221	1.222

Abbreviations: CF, crude fiber content; CFat, crude fat content; CPr, crude protein; GE, gross energy content; MC, moisture content; SE, standard error; TA, total ass content; UCa, utilizable carbohydrate.

#### 3.5.8 Morphological characteristics of okra

**FIGURE 8:**
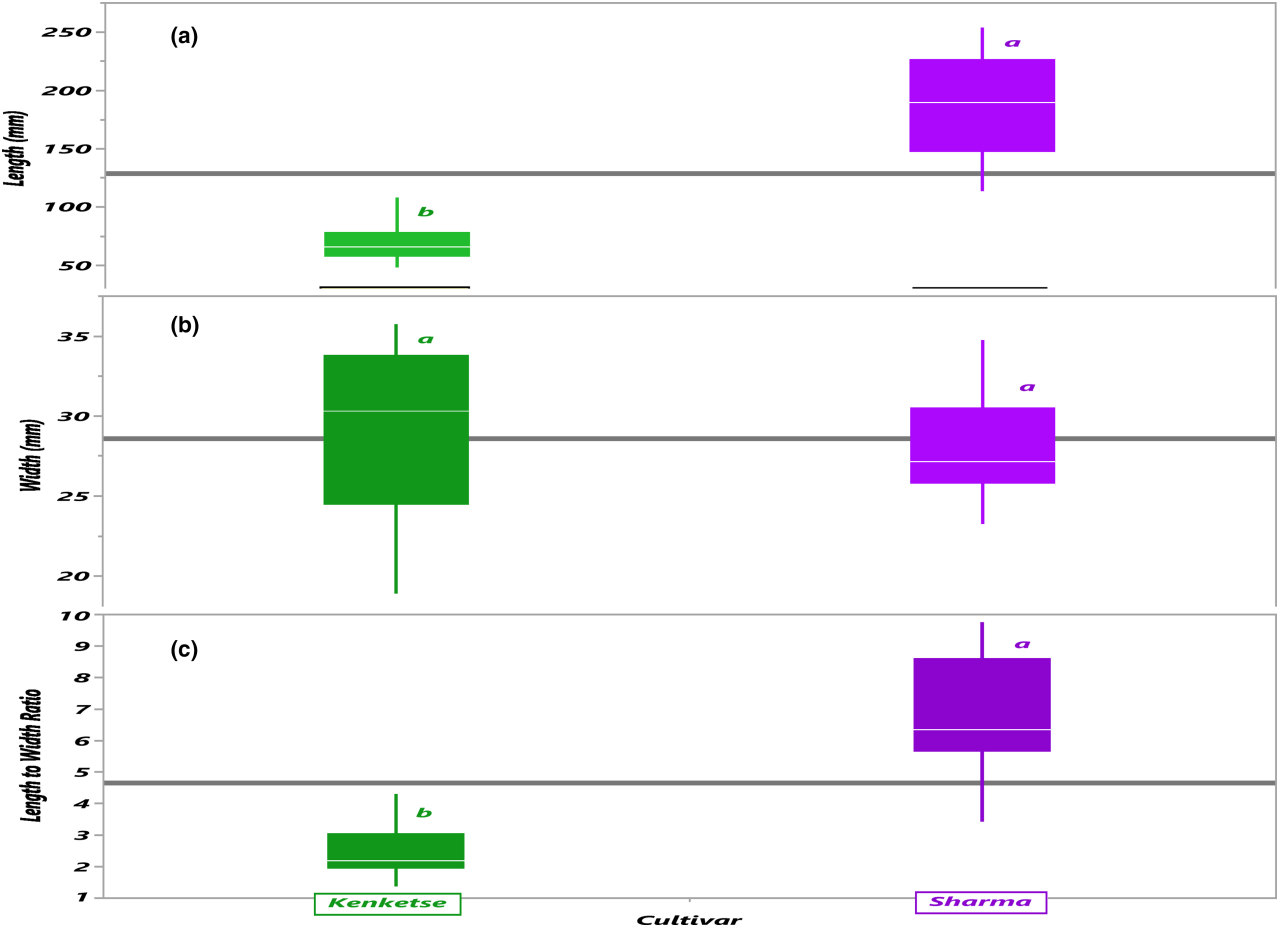
Geometric physical characteristics of two okra pods, representing their length (a), width (b), and length‐to‐width ratio (c).

The morphological characteristics of Kenkeste and Sharma pods (length and width) were significantly different (Table 7). The mean length and width of the Kenketse pod were 70.06 and 29.142 mm, respectively, whereas those of Sharma were 187.078 and 28.041 mm. The mean length and width of Kenketse are comparable to the mean length and width of okra pods recorded by Davies (2020). Sharma pod had a substantially longer mean length than Kenketse. Although there is no significant difference in width, the difference in pod length between the two okra cultivars is substantial (Table 7). Sharma also had a higher length‐to‐width ratio (Figure 8). There are similar reports from previous studies (Abdalla et al., 2023; Davies, 2020). These variations in morphological traits might have an impact on proximate compositions and attributes. Altogether, the proximate compositions and morphological traits of the two okra varieties differ profoundly.

**TABLE 7:** Morphological characteristics of pods of okra cultivars.

Cultivar	Variable	No. of sample	Pod	
			Mean	Standard devi.
Kenketse	Length	17	70.066 mm (7.06 cm)	4.589
	Width		29.142 mm (2.91 cm)	5.079
Sharma	Length	17	187.078 mm (18.7 cm)	10.983
	Width		28.041 mm (2.8 cm)	3.309

## 4 CONCLUSION AND RECOMMENDATION

Okra is an underutilized crop grown for its medicinal, nutritional, and economic value, and it is one of the indigenous vegetables in Ethiopia. The results unveiled the relative importance of okra in ethnomedicine, food, and nutrition. The cultivars are widely utilized in traditional medicine, for example, to treat gastrointestinal illnesses, diabetes, constipation, heart discomfort, wounds and cuts, diarrhea, back pain, and amoebae. The entire parts in this regard are utilized to treat various ailments in the research areas. Furthermore, the pods of the two cultivars had a high moisture content and were excellent sources of crude protein, crude fat, crude fiber, utilizable carbohydrate, and gross energy, all of which contribute significantly to the enrichment of the human diet. Specifically, Kenketse pods contained a substantially higher quantity of proximate components when compared to the pods of Sharma. In terms of morphological traits, Kenketse and Sharma differed greatly in pod dimensions, with Sharma having significantly longer pod length than Kenketse and Kenketse having significantly wider pod width than Sharma. The variations in the morphological characteristics could account for the variations in their proximate compositions. Collectively, the rich medicinal benefits, food value, and nutritional profile of okra will aid in the diversification of traditional medicine and the monotonous eating habits of the local communities. Therefore, promotion of production and consumption of okra could help alleviate food insecurity and malnutrition among the local communities, thereby helping to realize the full potential of this versatile, and underutilized indigenous vegetable.

## AUTHOR CONTRIBUTIONS

All authors participate in problem identification, data collection, data analysis, as well as write up and revision of the manuscript.

## FUNDING INFORMATION

This study was funded by the Office of Vice President for Research and Technology Transfer at Hawassa University, Ethiopia.

## CONFLICT OF INTEREST STATEMENT

The authors declare no conflicts of interests.

## Data Availability

All the data used for this study are already included in the manuscript itself.
